# Amiodarone-induced reversible and irreversible hepatotoxicity: two case reports

**DOI:** 10.1186/s13256-018-1629-8

**Published:** 2018-04-14

**Authors:** Toyonobu Tsuda, Hayato Tada, Yoshihiro Tanaka, Naoto Nishida, Taiji Yoshida, Takeshi Sawada, Kenji Sakata, Kenshi Hayashi, Masa-aki Kawashiri, Takeru Oyama, Motoko Sasaki, Nozomu Kurose, Masakazu Yamagishi

**Affiliations:** 1 Division of Cardiovascular and Internal Medicine, Kanazawa University Graduate School of Medical Sciences, 13-1 Takara-machi, Kanazawa, 920-8640 Japan; 2 Department of Molecular and Cellular Pathology, Kanazawa University Graduate School of Medical Sciences, Kanazawa, Japan; 3 Department of Human Pathology, Kanazawa University Graduate School of Medical Sciences, Kanazawa, Japan; 4 Department of Pathology and Laboratory Medicine, Kanazawa University Graduate School of Medical Sciences, Kanazawa, Japan

**Keywords:** Amiodarone, Hepatotoxicity

## Abstract

**Background:**

Amiodarone is a highly effective treatment for supraventricular and ventricular tachyarrhythmia; however, it could be associated with several serious adverse effects, including liver injury.

**Case presentation:**

We report the clinical and histological features of two contrasting Japanese patients with amiodarone-induced reversible and irreversible hepatotoxicity. One patient with amiodarone-induced irreversible hepatotoxicity showed liver cirrhosis during treatment with amiodarone and died of hepatic failure; the other patient, who had reversible hepatotoxicity, showed a reversible course of liver function and imaging after discontinuation of amiodarone.

**Conclusions:**

We emphasize the importance of close monitoring of liver enzymes and evaluation of liver computed tomographic imaging as well as liver biopsy during treatment with amiodarone, and discontinuation should be considered when amiodarone-induced hepatotoxicity is suspected.

## Background

Amiodarone is a highly effective treatment for supraventricular and ventricular tachyarrhythmia; however, it could be associated with several serious adverse effects, including pulmonary toxicity [1], thyroid dysfunction [2], and liver injury [3]. Serious adverse effects sometimes result in a poor prognosis because of the very long plasma half-life of amiodarone. Amiodarone-induced hepatotoxicity can progress to cirrhosis, resulting in decompensated hepatic failure, although this rarely happens [4]. In this report, we present the clinical features of two patients with amiodarone-induced reversible and irreversible hepatotoxicity, highlighting the importance of close monitoring of liver enzymes and evaluation of liver computed tomographic (CT) images as well as liver biopsy during treatment with amiodarone.

## Case presentation

### Patient 1

**Fig. 1:**
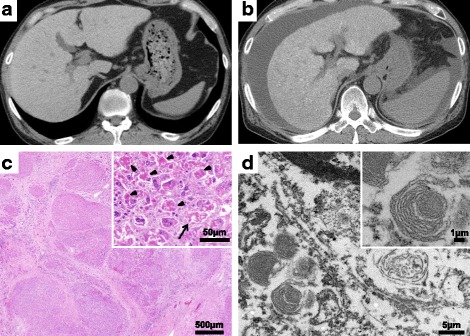
**a** Computed tomographic scan shows diffuse high attenuation of the liver parenchyma (96 Hounsfield units). **b** Massive ascites and splenomegaly were found, in addition to diffuse high attenuation of the liver parenchyma. **c** Regenerative nodules and well-developed bridging fibrosis were observed (hematoxylin and eosin stain, magnification × 20), as were marked neutrophilic infiltration, a remarkable amount of Mallory bodies (*arrowheads*), and ballooned hepatocytes (*arrow*) (hematoxylin and eosin stain, magnification × 200). **d** Numerous whorled or lamellar inclusions in lysosomes were detected by electron microscopy

Patient 1 was a 62-year-old Japanese man admitted to our hospital for general malaise and systemic edema. He had been an office worker, had a history of smoking, and had no history of alcohol intake. He had no apparent family history of cardiovascular and liver diseases. He had been diagnosed with cardiac sarcoidosis complicated with ventricular tachycardia (VT) when he was 49 years old. Since then, he had been treated with corticosteroids (prednisolone 10 mg/day) and amiodarone (150 mg/day), in addition to enalapril (5 mg/day), metoprolol (20 mg/day), and lansoprazole (15 mg/day). After the initiation of amiodarone, the patient’s liver enzymes were gradually as well as intermittently exacerbated to elevated over the course of 13 years (peak aspartate aminotransferase 161 IU/L [normal range 9–32 IU/L]; peak alanine aminotransferase 106 IU/L [normal range 4–37 IU/L]); however, the treating physician decided to continue amiodarone to control the patient’s VT. During the follow-up period, the patient’s mean plasma level of amiodarone was 1.8 μg/ml and that of desethylamiodarone was 1.1 μg/ml. An abdominal plain CT scan showed diffuse high attenuation of the liver parenchyma (Fig. 1a). The patient had no history of hospitalization for heart failure after the introduction of amiodarone. However, general malaise and systemic edema occurred, and abdominal ultrasound showed liver cirrhosis, splenomegaly, and massive ascites.

At admission, the patient’s blood pressure was 95/66 mmHg, his heart rate was 69 beats/minute, and his body temperature was 35.6 °C. His consciousness was clear, and his respiratory sounds were normal. Marked distention of the abdomen was observed, and the liver and spleen were palpable from the abdominal wall. No abnormal neurological finding was observed.

A chest x-ray revealed mild cardiomegaly (cardiothoracic ratio 50%) without marked congestion and pleural effusion. Echocardiography showed mild left ventricular systolic dysfunction (ejection fraction 45%) and mild mitral and tricuspid regurgitation without a finding of pulmonary hypertension. Blood tests showed thrombocytopenia (81 × 10^3^/μl), elevations of liver and ductal enzymes (aspartate aminotransferase 135 IU/L, alanine aminotransferase 91 IU/L, γ-glutamyl transpeptidase [γ-GTP] 202 IU/L, total bilirubin 2.4 md/dl), and hypoalbuminemia (serum albumin 3.1 g/dl). The patient’s brain natriuretic peptide level was mildly elevated (136 pg/ml), which showed no change during follow-up. Other serologic study results were negative for chronic viral hepatitis and other liver diseases. An abdominal plain CT scan showed massive ascites and splenomegaly, in addition to diffuse high attenuation of the liver parenchyma (Fig. 1b). At that time, amiodarone was discontinued because amiodarone-induced hepatotoxicity was suspected. However, the patient died of hepatic insufficiency during hospitalization. An autopsy revealed yellow liver specimens with a rough surface that suggested cirrhotic changes. Hematoxylin and eosin staining showed regenerative nodules and well-developed bridging fibrosis. Marked neutrophilic infiltrates, a remarkable amount of Mallory bodies, and hepatocellular ballooning were observed (Fig. 1c). Moreover, electron microscopic examination detected numerous whorled or lamellar bodies in the lysosomes (Fig. 1d). These findings helped diagnose amiodarone-induced hepatotoxicity associated with irreversible liver cirrhosis.

### Patient 2

Patient 2 was a 74-year-old Japanese man who underwent hemodialysis for chronic renal failure and was admitted to our hospital because of asymptomatic liver enzyme elevation. He had been working for the government, had never smoked, and had no history of alcohol abuse. He had no apparent family history of cardiovascular or liver diseases. At admission, his blood pressure was 126/52 mmHg, his heart rate was 46 beats/minute, and his body temperature was 36.4 °C. Physical and neurological examinations yielded no abnormal findings. Blood tests showed anemia (hemoglobin 11.5 g/dl), elevations of liver and ductal enzymes (aspartate aminotransferase 189 IU/L, alanine aminotransferase 150 IU/L, γ-GTP 1009 IU/L), elevation of C-reactive protein (1.04 mg/dl), and renal dysfunction (creatinine 3.34 mg/dl at 1 day after his regular hemodialysis). A chest x-ray revealed no abnormal findings. Echocardiography showed mild left ventricular systolic dysfunction (ejection fraction 48%) without any apparent structural diseases.

**Fig. 2:**
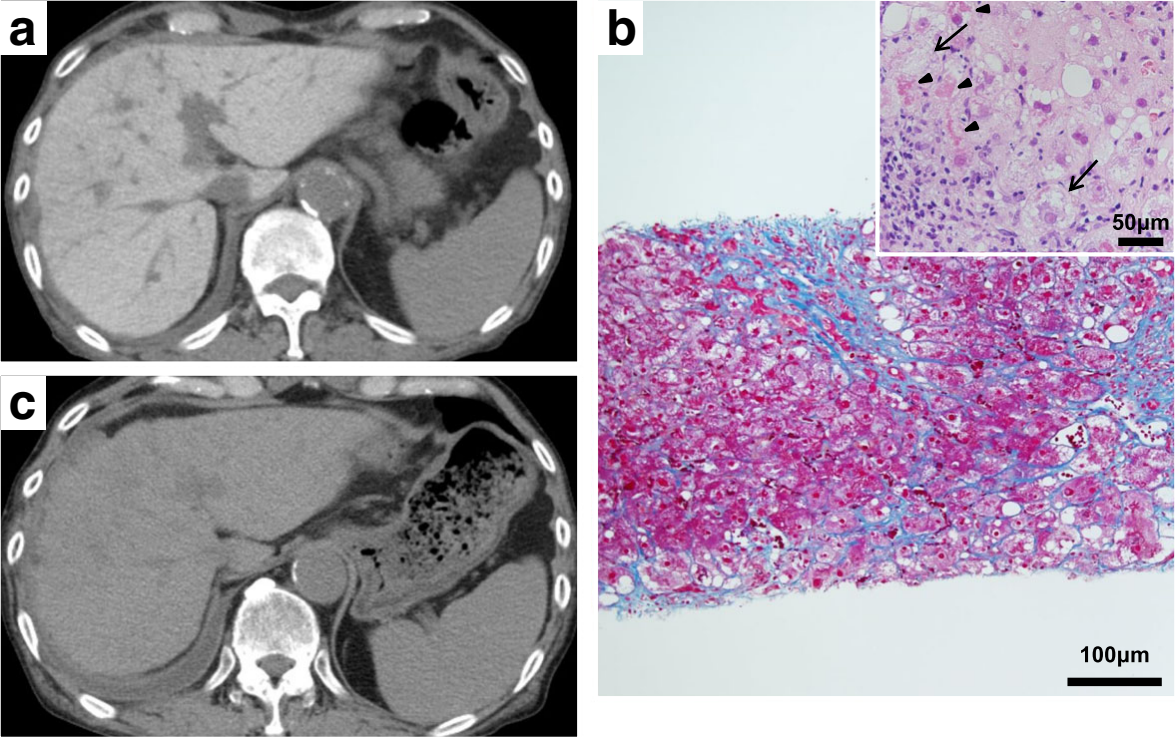
**a** Computed tomogram shows diffuse high attenuation of the liver parenchyma (120 Hounsfield units). **b** Distinct collagen deposition is seen in the periportal, perivenular, and pericellular locations, which formed bridging fibrosis (Azan stain, magnification × 100). There were numerous Mallory bodies (*arrowheads*) as well as hepatocellular ballooning (*arrows*) and mild macrovesicular and microvesicular fatty changes. Mild lymphocytic and neutrophilic infiltration was also observed (hematoxylin and eosin stain, magnification × 400). **c** After discontinuation of amiodarone, the patient’s liver density dramatically improved to a normal level (45 Hounsfield units) during the course of 9 months

Amiodarone (200 mg/day), in addition to enalapril (5 mg/day), spironolactone (25 mg/day), bisoprolol (2.5 mg/day), and lansoprazole (15 mg/day), was introduced when the patient was age 71 because of ventricular arrhythmia, including sustained VT, although no structural heart disease was observed. After the introduction of amiodarone, no ventricular arrhythmia was documented during follow-up. However, the patient’s liver enzymes were gradually exacerbated to elevated after beginning amiodarone over 1 year (peak aspartate aminotransferase 189 IU/L, peak alanine aminotransferase 150 IU/L). He had no clinical or laboratory evidence of congestive heart failure or other liver diseases. An abdominal plain CT scan showed diffuse high attenuation of the liver parenchyma, as seen in patient 1 (Fig. 2a). To investigate the cause of liver dysfunction, a liver biopsy was performed. Histologically, the liver showed steatohepatitis with severe perivenular, pericellular, and chicken wire–like periportal fibrosis (Fig. 2b); numerous Mallory bodies; hepatocellular ballooning; and mild macrovesicular and microvesicular fatty changes (Fig. 2b). Mild lymphocytic and neutrophilic infiltration was also observed. Bridging fibrosis divided the hepatic parenchyma, but regenerative nodules were not formed. These histological findings suggested amiodarone-induced nonalcoholic steatohepatitis. We decided to discontinue amiodarone. A cardioverter defibrillator implant was performed to prevent sudden cardiac death due to ventricular arrhythmia. After discontinuation of amiodarone, the patient’s plasma concentration of amiodarone gradually decreased from 1.4 to 0.07 μg/ml during 9 months, followed by improvement in liver enzymes. Notably, the patient’s liver enzymes and fibrosis markers, such as hyaluronic acid and type IV collagen, started to improve 2 months later, after the discontinuation of amiodarone. Under these conditions, a follow-up plain CT scan showed marked improvement of the liver parenchyma density (Fig. 2c). The patient’s liver enzymes decreased to within normal range 8 months after discontinuation of amiodarone.

## Discussion

In this report, we present the clinical features of two patients with amiodarone-induced reversible and irreversible hepatotoxicity, highlighting the importance of close monitoring of liver enzymes and evaluation of liver CT imaging as well as liver biopsy during treatment with amiodarone. Approximately 25% of patients using amiodarone develop a transient asymptomatic increase in serum aminotransferase levels that resolves spontaneously or after dose reduction [4]. Amiodarone-induced symptomatic hepatitis, cirrhosis, and fatal hepatic failure are extremely rare (0–3%) [4, 5]. However, when the diagnosis is established, the mortality risk may be as high as 60% at 5 months [6]. Histological features of amiodarone-induced hepatotoxicity are similar to alcoholic hepatitis and characterized by Mallory bodies, leukocyte infiltration, steatosis, and ballooning of hepatocytes [7]. Amiodarone and its metabolites accumulate in lysosomes of hepatocytes and lead to inhibition of phospholipase A1 and A2, which inhibits removal of lysosomal lipids and leads to phospholipidosis [8]. As with our patients, this mechanism leads to steatohepatitis and finally to irreversible liver cirrhosis. The presence of lamellar lysosomal inclusion bodies visualized by electron microscopy is a typical pathological finding of amiodarone-induced hepatotoxicity [7]. In patient 1, amiodarone was continued under the conditions of elevated liver enzymes in addition to the use of corticosteroids that could cause liver injury or accelerate his underlying liver injury, and it led to irreversible fatal hepatic failure. However, in patient 2, amiodarone was discontinued immediately after the detection of liver enzyme elevation and led to improvement of liver function. According to these two clinical courses, amiodarone should be discontinued immediately if amiodarone-induced liver injury is suspected.

Amiodarone is a lipophilic agent and tends to accumulate in lipid-laden organelles such as the liver. It is reported that a concentration of amiodarone and its metabolite, *N*-desethylamiodarone, in the liver may be as high as 500-fold of the serum level [9].

Previous reports showed that the plasma amiodarone level may be poorly correlated with its toxicity [10]. However, high liver density seen on CT scans, which is secondary to increased iodine content of amiodarone and reflects the tissue level of amiodarone, may be useful to detect the toxicity [11]. In this regard, it is important to evaluate elevations of liver enzymes, liver CT imaging (high attenuation of the liver parenchyma), and histology, and discontinuation should be considered when amiodarone-induced hepatotoxicity is suspected. Alternative strategies, such as catheter ablation and/or an implantable cardioverter defibrillator implant could be considered for such patients.

## Conclusions

Amiodarone-induced hepatotoxicity showed a reversible or irreversible course, depending on when or if the drug was discontinued. Close monitoring of liver enzymes and evaluation of liver CT imaging and liver biopsy results should be considered, and amiodarone should be discontinued when amiodarone-induced hepatotoxicity is suspected.
